# Differences in creep response of GBM cells migrating in confinement

**DOI:** 10.1080/23335432.2020.1757509

**Published:** 2020-05-26

**Authors:** Ishan Khan, Loan Bui, Robert Bachoo, Young-Tae Kim, Cheng-Jen Chuong

**Affiliations:** a Joint Graduate Program in Biomedical Engineering, University of Texas at Arlington and University of Texas Southwestern Medical Center at Dallas; b Department of Neurology & Neurotherapeutics University of Texas Southwestern Medical Center at Dallas, Dallas, Texas; c Department of Bioengineering, University of Texas at Arlington, Texas

**Keywords:** Cancer cell, glioblastoma, viscoelastic properties, creep, actomyosin contraction

## Abstract

Using a microfluidic platform to apply negative aspiration pressure (–20, –25, –30, –35 and –40 cm H_2_O), we compared the differences in creep responses of Glioblastoma Multiforme (GBM) cells while migrating in confinement and at a stationary state on a 2D substrate. Cells were either migrating in a channel of 5 x 5 μm cross-section or stationary at the entrance to the channel. In response to aspiration pressure, we found actively migrating GBM cells exhibited a higher stiffness than stationary cells. Additionally, migrating cells absorbed more energy elastically with a relatively small dissipative energy loss. At elevated negative pressure loads up to – 30 cm H_2_O, we observed a linear increase in elastic deformation and a higher distribution in elastic storage than energy loss, and the response plateaued at further increasing negative pressure loads. To explore the underlying cause, we carried out immuno-cytochemical studies of these cells and found a polarized actin and myosin distribution at the front and posterior ends of the migrating cells, whereas the distribution of the stationary group demonstrated no specific regional differences. These differences in creep response and cytoskeletal protein distribution demonstrate the importance of a migrating cell’s kinematic state to the mechanism of cell migration.

## Introduction

Every year approximately 5 out of every 100,000 individuals are diagnosed with primary malignant brain tumors and 80% of these diagnosed are cases of malignant gliomas (Alifieris and Trafalis 2015). Glioblastoma Multiforme (GBM), known as the most common glioma among all adult primary brain tumors, consists of a heterogeneous population of cells within the tumor mass (Yuan et al. 2004). The median survival of patients diagnosed with GBM is only 15 months (Alifieris and Trafalis 2015) and even after a complete surgical resection (if feasible) in combination with chemotherapy and radiotherapy recurrence of the tumor has been observed in patients (Holland 2000). Invasiveness of peripheral GBM cells migrating from the primary tumor cite to different locations of the brain has been one of the major roadblocks in treating GBMs. Different from other cancer cell types, GBMs do not intravasate to the blood stream, but migrate in 3D confined space along the myelinated axonal fiber tracts of the white matter or the outer wall of blood vessels (Bernstein and Woodard 1995; Giese and Westphal 1996, G Gritsenko et al. 2012). This distinctive feature makes the migration dynamics of GBMs unique, signifies the role of active migration through narrow extracellular pathways in the metastasis of brain tumor.

Studies have shown that to migrate through confined space of extracellular matrix (ECM), the cells undergo a remodeling process, with biophysical property changes that facilitate the migration process. Changes in migration phenotype include velocity, directionality, and persistence, among others (Irimia and Toner 2009; Pathak and Kumar 2012). Mechanical interaction of a cell with microenvironment and its responses can play a major role in the development of invasive phenotype of cancer cells. Quantitative assessment of how cells respond to external mechanical stimuli can provide insight into their migration biomechanics.

Studies have demonstrated that deformability of cancer cells has a direct correlation with their invasiveness (Ochalek et al. 1988; Igawa et al. 2004; Zhao et al. 2006, Ketene et al. 2012). These studies, conducted using different cell types on 2D substrates, showed a cancerous cell is more compliant than that at healthy state. While migrating in confined 3D microenvironment, a cancer cell can exhibit different mechanical phenotypes to meet its functional needs. A better understanding of how it mechanically interacts with the microenvironment can help us to better understand the biomechanics of the invasive progression of cancer cells.

In this work, we report the differences in the mechanical phenotypes of GBMs at two different states. We examined their differences in creep responses to a sudden application of negative aspiration pressure when they were either actively migrating in a confined channel of 5 × 5 µm cross section or in a stationary state at the entrance region to the confined channel from an open substrate. To correlate the observed differences in creep responses of GBMs in different states, we immuno-cytochemically treated the cells immediately after the creep study to examine their respective intracellular distributions of actin and myosin using fluorescence microscopy.

## Methods

### Fabrication of PDMS Device

Using fabricated microfluidic devices with narrow channels as the model platform, we studied GBMs migration in confinement. PDMS (Dow Corning, Sylgard 184) devices with microchannel of 5 × 5 µm cross section and a length of 530 µm connecting two reservoirs each at 100 µm height were fabricated using photo and soft lithography technique (Bui et al. 2017). A silicone elastomer base was mixed with a curing agent at a mixing ratio of 10:1. Reservoirs of 8 and 6 mm diameter were punched at the PDMS block to allow cell seeding and supply of nutrients. After sterilization, we coated the channel surfaces with laminin (Sigma-Aldrich) at 10 μg/ml prior to seeding cells. To maintain an isolated fluid environment, we plasma-treated the device and a glass cover slip for the device assembly (PSD Pro Series, Novascan).

### Cell Culture

Patient-derived CD 133^+^ GBM cells, provided by the University of Texas Southwestern Medical Center with IRB approval, were used. The cells were maintained in serum-free Dulbecco’s Modified Eagle Medium/F-12 medium (DMEM/F-12) with 2% B-27 (Invitrogen), 0.25% insulin-transferrin-selenium-X (Invitrogen), gentamicin at 25 μg/ml, and mouse EGF at 20 ng/ml prior to the study.

### Identification of GBMs in Two Groups: Actively Migrating and Stationary State

**Figure 1.**
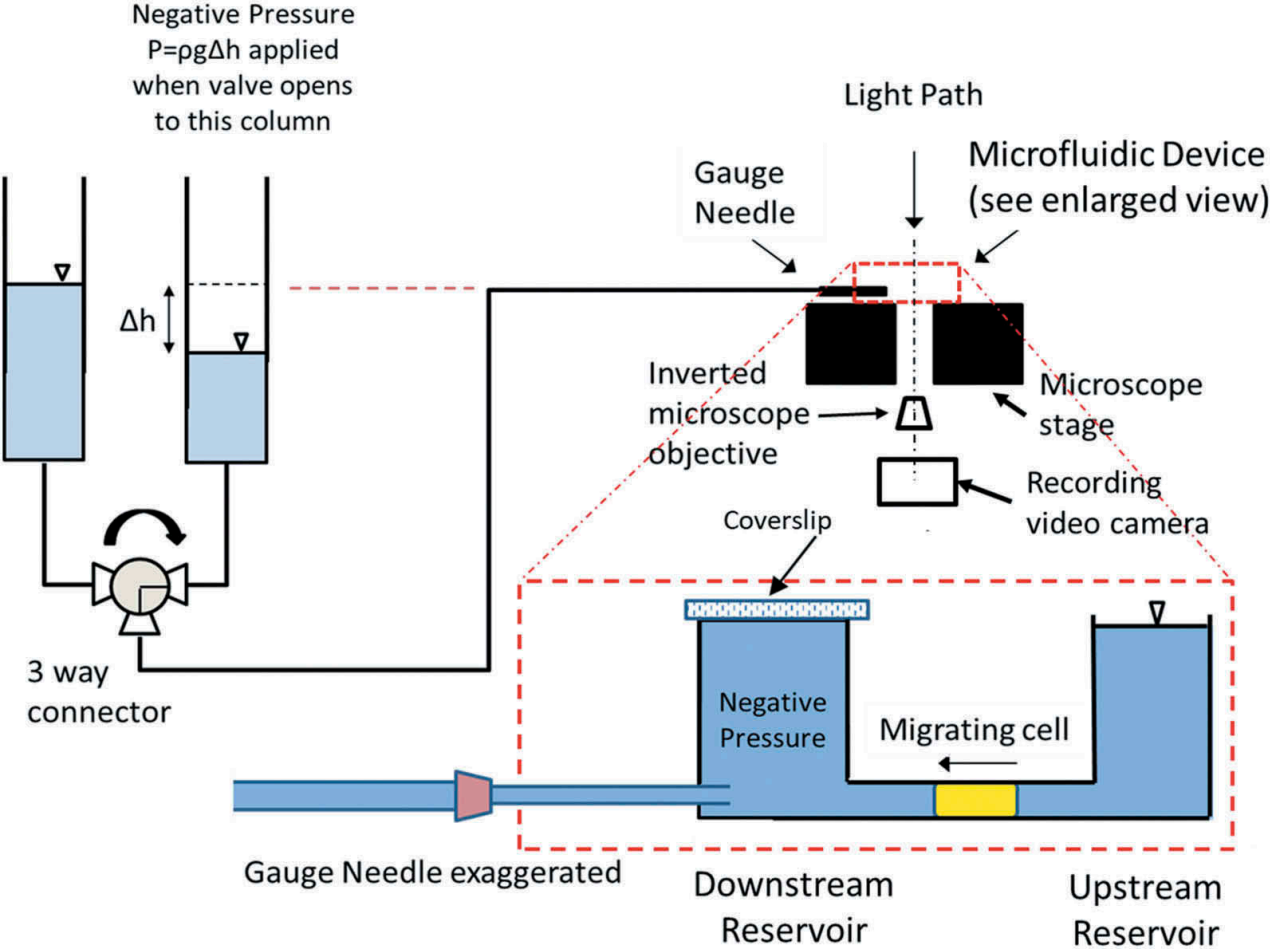
Schematic of the experimental setup with enlarged view of the microfluidic device placed on the stage of an inverted microscope. After cell seeding to the bottom surface of the upstream reservoir near the channel, cells migrated into the 5 × 5 µm channel as illustrated. Using a three-way valve, we delivered the prescribed negative pressure to the front-end of the migrating cell through a gauge needle inserted through the wall of the downstream reservoir. The time course of the displacements at the front-end of the cell was measured from recorded video images. Note that, we sealed the pressure at the downstream reservoir right before we activated the negative pressure application using a cover slip

The GBMs were seeded close to the channel openings to the upstream reservoir (Figure 1) in a density of 500,000 cells/120 µL. Following the seeding, we monitored the cell motility at least every 6 h for 36 h. Cells that had reached, stopped at the channel opening and blocked the opening cross section were identified as in ‘stationary’ state. Cells migrated to and entered channels demonstrated intermittent migration in the channel. Those that had reached at least halfway of the channel length (~ 265 µm) were identified as in ‘actively migrating’ state. Creep test was performed on GBMs in these two states wherein we examined their differences in deformation characteristics to the sudden application of prescribed pressure load through the microchannel.

### Experimental setup

VTwo water columns connected to a three-way valve delivered either the prescribed negative pressure or the baseline atmospheric pressure to the media in the downstream reservoir and the microchannel. Figure 1 with its enlarged view of the PDMS device (in red-dashed line box) illustrates the delivery of the pressure load from the source column through a gauge needle to the cell in the channel. In migrating group, cells had actively migrated half way in the channel. Whereas cells in stationary group practically blocked the channel near the channel entrance for at least 30 min with large portion of the cell body adhered to the bottom surface of the upstream reservoir without any noticeable movement.

Prior to the application of aspiration pressure, we sealed the open surface of the downstream reservoir with a glass cover slip coated with film forming acrylate solution known to be non-cytotoxic (Vallittu and Ekstrand 1999). The prescribed aspiration pressure was applied to the front-end of the cell instantaneously when we opened the three-way valve to the column with matched water level (Figure 1). A Leica inverted microscope with 20x objective lens recorded the transient deformation of the cell at a framing rate of 19 fps using software *Leica* and *My Screen Recorder* (Deskshare Inc., NY). For both groups, we applied prescribed negative pressure for 30 s, released it to zero baseline at 40 s. Deformation at the front-end of the cell was measured frame-by-frame using *Image J* (https://imagej.nih.gov/ij/).

### Creep Responses – Actively migrating vs stationary

**Figure 2.**
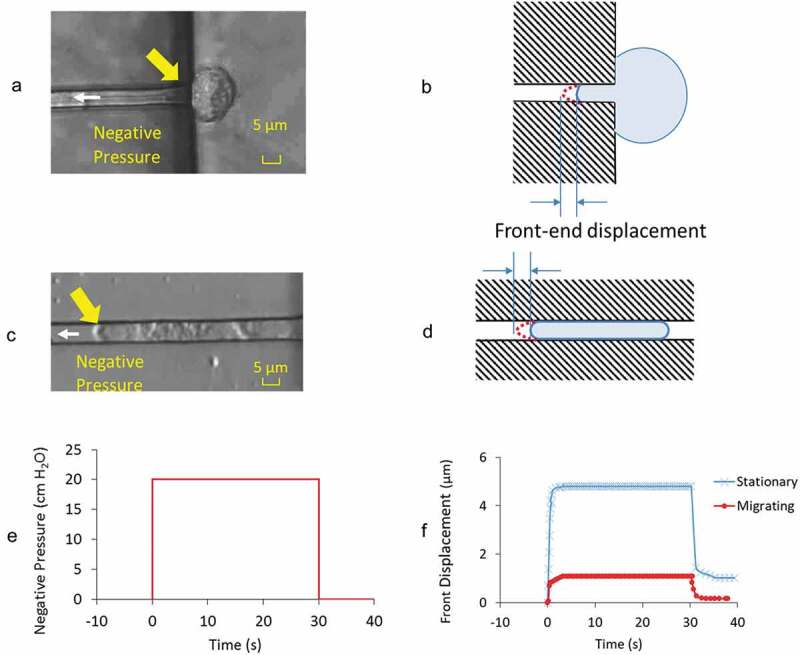
Images showing the front-end displacement of a GBM cell in A) stationary and C) actively migrating state under applied negative pressure of – 20 cm H_2_O at the direction of white arrow head. Yellow arrows highlight the front-end of the cell where measurements were taken. B) and D) are the respective illustrations to highlight the cell in microchannel and their front-end displacement at the loaded state. E) Prescribed negative pressure was activated, maintained at constant level for 30 s before its release to baseline value. F) Representative front-end displacements from the cell in stationary and migrating state through the time course of negative pressure application

Figure 2 A,C are video images of the GBMs from stationary and actively migrating groups when under the application of – 20 cm H_2_O aspiration pressure. Figure 2E shows the time course of pressure application with an instantaneous rise to full value at time = 0 s followed by its release at time = 30 s. The front-end displacements are the instantaneous position of the leading tip of the cell membrane relative to its initial position (Figure 2B, D) accounting for the collective contributions from the underlying actin cortex, regional cytoskeletal protein networks, their activities in the cytosol. Measurements revealed the differences in the deformation characteristics of GBMs’ creep response in two different states (Figure 2F).

**Figure 3.**
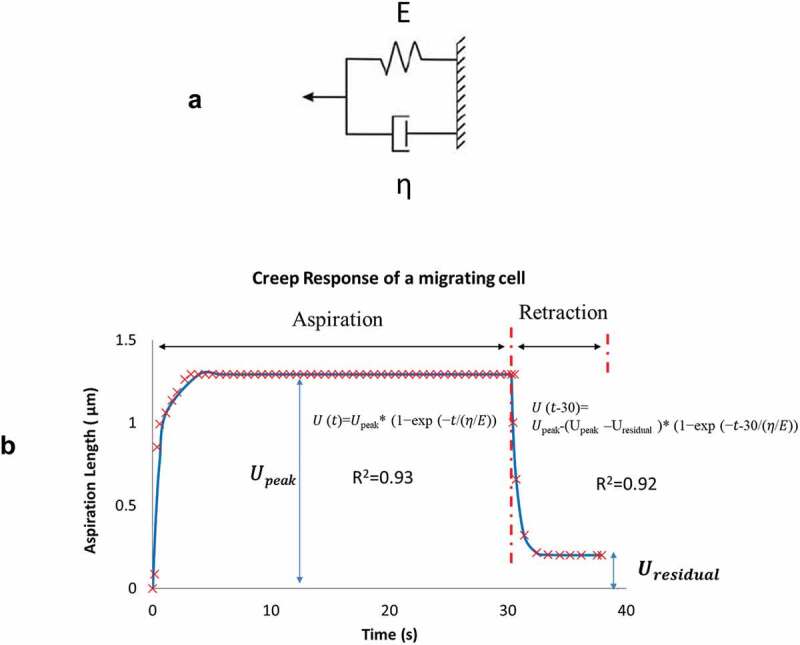
A) A Voigt model representation that consists of an elastic spring (spring constant *E*) and a dashpot (damping coefficient *η*) connected in parallel. B) Measurements of recorded front-end displacements was divided into two phases: aspiration from 0 to 30 s and retraction from 30 to 40 s. Each phase was curve-fitted to a Voigt model representation as shown

Measurements of the front-end displacement *U(t)* were fitted to a Voigt model (Fung and Fung 1977) that consists of an elastic spring (constant *E*) and a viscous damper (damping coefficient *η*) connected in parallel (Figure 3A). For each cell, we fitted the displacement data to the model in two separate phases: 1) aspiration phase in response to the instantaneous negative pressure application from 0 to 30 s, and 2) retraction phase that after the sudden release of the negative pressure from 30 to 40 s.

Aspiration:

(Eq. 1) $$U(t)\;\;=\;\;\;U_{peak}*\;\;\;\left(1-exp^{-\;\;\;\frac{t}{\left(\frac{\eta }{E}\right)}}\right)\;\;\;for\;0\leq t\;\;\leq \;\;30sec$$

Retraction:

(Eq. 2) $$U(t-30)=U_{peak}-\left(U_{peak}\;-\;U_{residual}\right)*\;\;\left(1-exp^{-\;\;\;\frac{t-30}{\left(\frac{\eta }{E}\right)}}\right)\;for\;t>30sec$$

Data fitting was done by finding *η/E* and *U_peak_* (*or U_residual_* for retraction) that minimize the objective function *OBJ* defined as the sum of the normalized difference between the fitted displacement and the measurements:

(Eq. 3) $$OBJ=\left(\frac{1}{N}\right)\;\;\;\sum_{n=1}^{N}\;\;\left[\frac{U_{expt}(t_{n})\;\;-\;\;U_{Voigt}(t_{n})}{U_{expt}(t_{n})}\right]$$

where *N* denote the total number of data points. Using generalized reduced gradient regression method **GRG2** (*Microsoft Excel & Frontline Systems Inc., NV*), fitted parameters *η/E* and *U*_peak_ (*or U*_residual_ for retraction) were identified as the pair that minimized the objective function with a tolerance for convergence set at < 0.001. The creep response in two phases and their respective model parameters *η/E* and *U*_peak_, *U*_residual_ are illustrated in Figure 3B.

At each pressure level, we compared the creep response parameters *U*_peak_ (or *U*_residual_) and *η/E* between the actively migrating and stationary group for the aspiration and the retraction phase. To examine any nonlinear response at elevated negative pressure load, we repeated experiment at −20, −25, −30, −35 and −40 cm H_2_O.

#### Any sliding movement of the cell in the channel or intracellular fluid movement?

To ensure the recorded cellular front-end displacements are the intrinsic deformation of the cell to the aspiration pressure, excluding any sliding movement arises from cell dislodgment from the channel wall, we measured the displacement at the corresponding posterior-end of the same cell to allow assessment of any sliding movement.

Cells in stationary group were in a stalling state with a portion of cell body blocking the channel entrance and the rest adhering to the bottom surface of the upstream reservoir. Video images show shrinkage of the lamellipodium on the substrate, suggesting cytoplasma flow from the rear (on 2D substrate) to the front protrusion (in the channel) compartment of the cell at the application of aspiration pressure. In addition to the displacement of the posterior-end toward the front, we assessed the degree of this cytoplasma movement and discussed how they could affect the measurement of aspiration length and the cellular creep responses.

We first determined the area of the cell adhering to the substrate before and after the aspiration pressure application from video images. With the assumption, the area approximates a segment of a circle, we estimated their effective radii (e.g., see images of Figure 7A and illustration of Figure 8C). We then estimated the volume loss in cytoplasma from the lamellipodium shrinkage along the outside perimeter of the cell (*∆V_rear_*) by using the area decrease and an estimated lamellipodium thickness *H_avg_* from

(4) $$\Delta V_{rear}=\left\{\frac{2}{5}\pi \left(R^{2}-R_{shrink}^{2}\right)\right\}*H_{avg^{.}}$$

where *R* and *R_shrink_* are the effective radii before and after shrinkage through the process. The factor of 2/5 π was used to account for the effective radians of the fitted circular segment. The volume loss was considered to partially contribute to a volume increase at the front protrusion **Δ***V_front_* and a corresponding effective aspiration length $\Delta {\bar{U}}_{effective}$. We thus calculated the effective aspiration length $\Delta {\bar{U}}_{effective}=\frac{\Delta V_{front}}{5\;\text{\textbackslash{}mum}\;x\;5\;\text{\textbackslash{}mum}}$ and subtracted it from the apparent aspiration length measurement for all cells in stationary group. Using this approach, we calculated the effect of lamellipodium shrink on the apparent aspiration length measurement.

#### Immuno-cytochemistry of filamentous Actin and Myosin

To examine the differences in actin and myosin distributions between migrating and stationary groups, we immuno-stained filamentous actin, myosin, and nucleus of the GBMs. Immediately after the creep test, we removed the coverslip from the downstream well, drained the culture media, fixed the cell with 4% paraformaldehyde in 1× PBS at 4°C for 10 min. Following the removal of paraformaldehyde, the device was washed with 1× PBS three times. To aid visualization of cell nucleus, 7 *μL* of 4′,6-diamidino-2-phenylindole (DAPI) dissolved in 10 mL of solution (0.5% triton in 1× PBS) was introduced. For visualization of myosin filaments, samples were blocked in goat serum for 1 h followed by staining with rabbit anti-myosin IIa (#3403, 1:50, Cell Signaling Technology) overnight at 4°C. The following day, samples were washed with 1xPBS three times, incubated with Alexa Fluor® 594 AffiniPure goat anti-rabbit IgG (1:200, Jackson Immuno Research Lab.) at room temperature for 2 hrs. For visualization of actin filaments, samples were stained with Actin-stain^TM^ 488 (3:500, #PHDG1, Cytoskeleton) at room temperature for 2 h. All expression were visualized using fluorescence microscopy (Zeiss Observer Z1) with images captured using 20 X objective.

#### Statistical Treatment

A non-parametric two-tailed *t*-test (Excel, Microsoft) was used to compare aspiration lengths *U*_peak_ (or residual lengths *U*_residual_) and *η/E* ratios between the migrating and the stationary groups for data from both the aspiration and the retraction phases. We considered the differences in creep response parameters to be significant if *p* < 0.05.

## Results

### Creep responses at two different states

Aspiration phase

**Figure 4.**
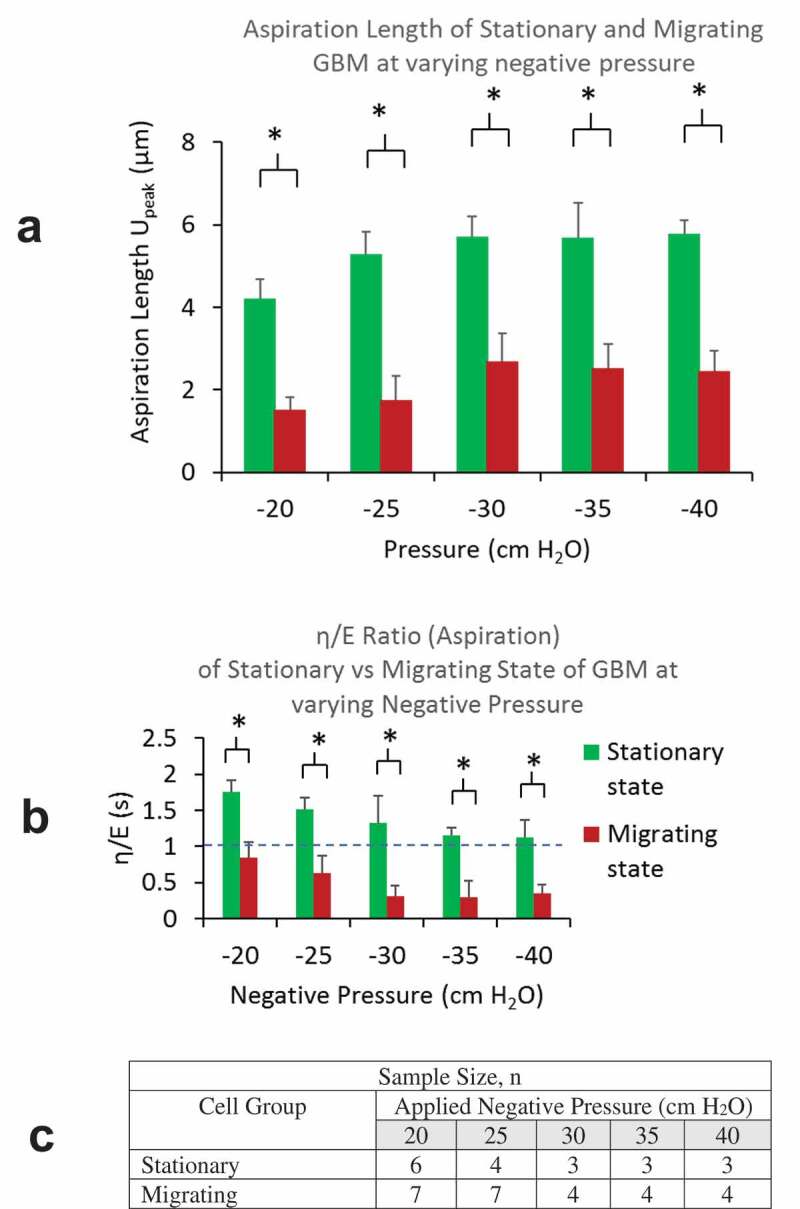
A) Aspiration lengths of cells (*U*_peak_) in migrating group were found to be much lower than that in stationary groups for all negative pressure load from −20 to −40 cm H_2_O. B) *η/E* ratios of cells in migrating group were found to be less than 1 and much lower than that in stationary groups for all pressure load from −20 to −40 cm H_2_O. C) A table summarizing sample sizes from stationary and migrating groups at different pressure levels

Comparison of creep response parameters *U*_peak_ andη/Eratio at increasing level of negative pressure from the aspiration phase are presented in Figure 4A, B. For all pressure levels, cells in migration state were found to have much lower *U*_peak_, indicating their higher stiffness than those in stationary state. Further, cells in migrating state were found to have a much lower η/Eratio, being less than 1, indicating the relative dominance of the elastic response over the energy dissipation in their deformation response to load application. The table in Figure 4C summarizes the numbers of cells used in these results.

Results from both groups show approximately linear increase in *U*_peak_ at increasing aspiration pressure up to −30 cm H_2_O, beyond which the deformation appeared to reach a plateau (Figure 4A). The cells became much stiffer at higher negative pressure load with little increase in deformation for further load increase. Results also showed decreasing η/Eratio with negative pressure load from 0 to −30 cm H_2_O. The ratios flattened out as the negative pressure exceeds – 30 cmH_2_O. Reducing damping-to-elastic ratios is indicative of the progressively higher contribution of the elastic response and reducing fluid-like energy dissipation. At higher negative pressure load, the ratio in the distribution of elastic vs. viscous responses appear to remain at the same level (Figure 4B).

Retraction phase

**Figure 5.**
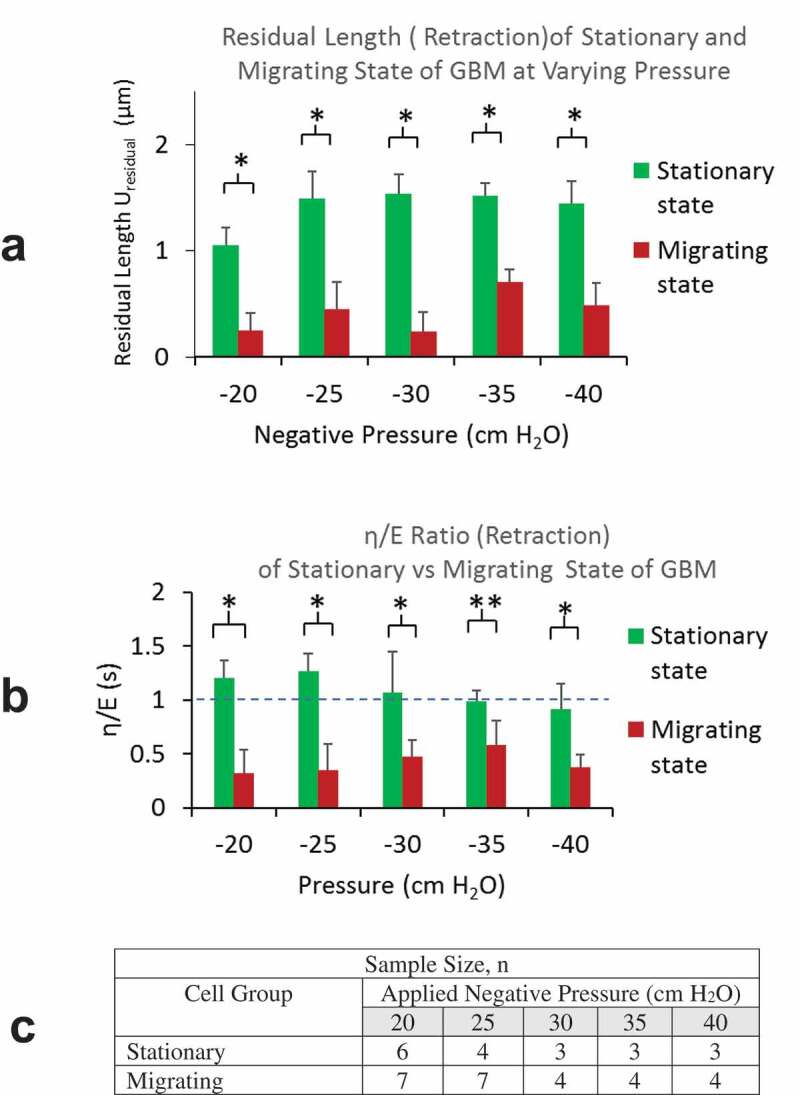
A) Residual lengths of cells (*U*_residual_) in migrating group were found to be much lower than that in stationary groups for all negative pressure load from −20 to −40 cm H_2_O. B) *η/E* ratios of cells in migrating group were found to be less than 1 and all much lower than that in stationary groups for all pressure load from −20 to −40 cm H_2_O. C) A table summarizing sample sizes from stationary and migrating group at different pressure levels

Residual length (*U*_residual_) accounts for the residual deformation after the release of the load. The magnitudes of η/Eratio is indicative of the rate of instantaneous recoil from the peak deformation immediately after the release of aspiration pressure load. For all pressure levels, we found smaller *U*_residual_ from cells in migrating group, indicating their favored elastic energy recovery. Higher *U*_residual_ were found from GBMs in stationary group, indicating their incomplete elastic recovery at the release of pressure load. From stationary group, we observed increasing *U*_residual_ with the level of aspiration pressure load from 0 to −25 cm H_2_O, reaching a plateau for higher aspiration pressures. The residual lengths *U*_residual_ from migrating GBMs were found to be significantly lower for all pressure levels, yet we were not able to see a clear trend in the lengths vs. the pressure levels (Figure 5A).

From migrating GBMs, lower values in η/Eratios (being < 0.5) indicated their rapid recoil that favors elastic recovery over dissipative frictional loss. From the stationary group, we found η/E ratios greater or slightly less than 1, suggesting their greater or comparable viscous loss over the elastic energy recovery (Figure 5B).

From Aspiration to Retraction Phase

We characterized the GBM’s creep response characteristics. Aspiration length *U_peak_* reflects the cell’s capacity to resist elastic deformation; whereas residual length *U_residual_* indicates the impairment in the elastic properties of the cell at the release of the load. Through the cycle, hysteresis develops due to energy loss. Fitted η/E ratios from retraction phase were seen to differ significantly than that from aspiration phase. We attributed the energy loss through hysteresis as part of the causes for the observed differences.

### Any sliding movement of the cells in the channel or intracellular fluid movement?

**Figure 6.**
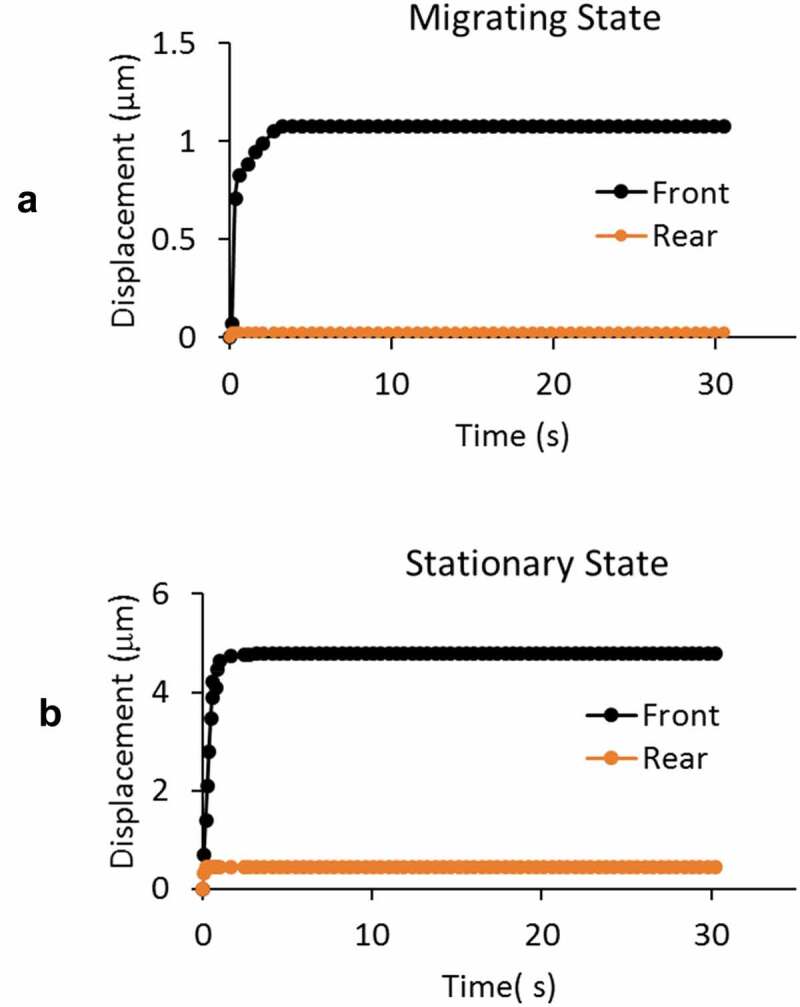
A) Simultaneous recording of the front and posterior-end displacements of a GBM cell in actively migrating state show near-zero displacement at the posterior-end, indicating the recorded front-end displacement are due to the cell deformation in response to the negative pressure load (at – 20 cmH_2_O), excluding any likely sliding movement of the cell in the channel. B) Cells from stationary group showed both front and posterior-end displacement. The corresponding intracellular fluid movement from the rear to the front compartments of the cell is discussed in the text

**Figure 7.**
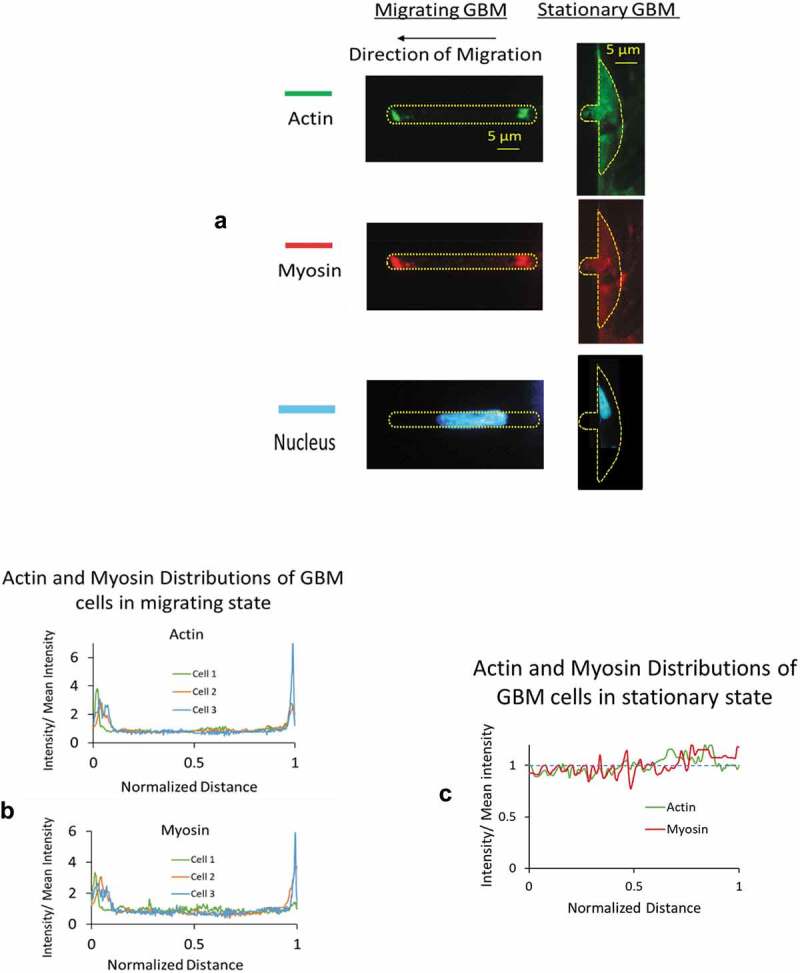
A) Immuno-cytochemical examination showed a significantly higher level of polarized actin and myosin-II filaments at both the front and posterior ends of cells in migrating group. Intracellular distributions from stationary group showed low level of expression without any regional preference. B) Polarized distributions in actin and myosin were found from GBM cells in migrating group. The fluorescent intensity along the axial length of the migrating cells was normalized with that of the entire cell. On the horizontal axis, 0 denotes the front end and 1 denotes the posterior end of the cells. C) From GBM cells in stationary group, distributions of normalized fluorescent intensities in both actin and myosin oscillate around 1

**Figure 8.**
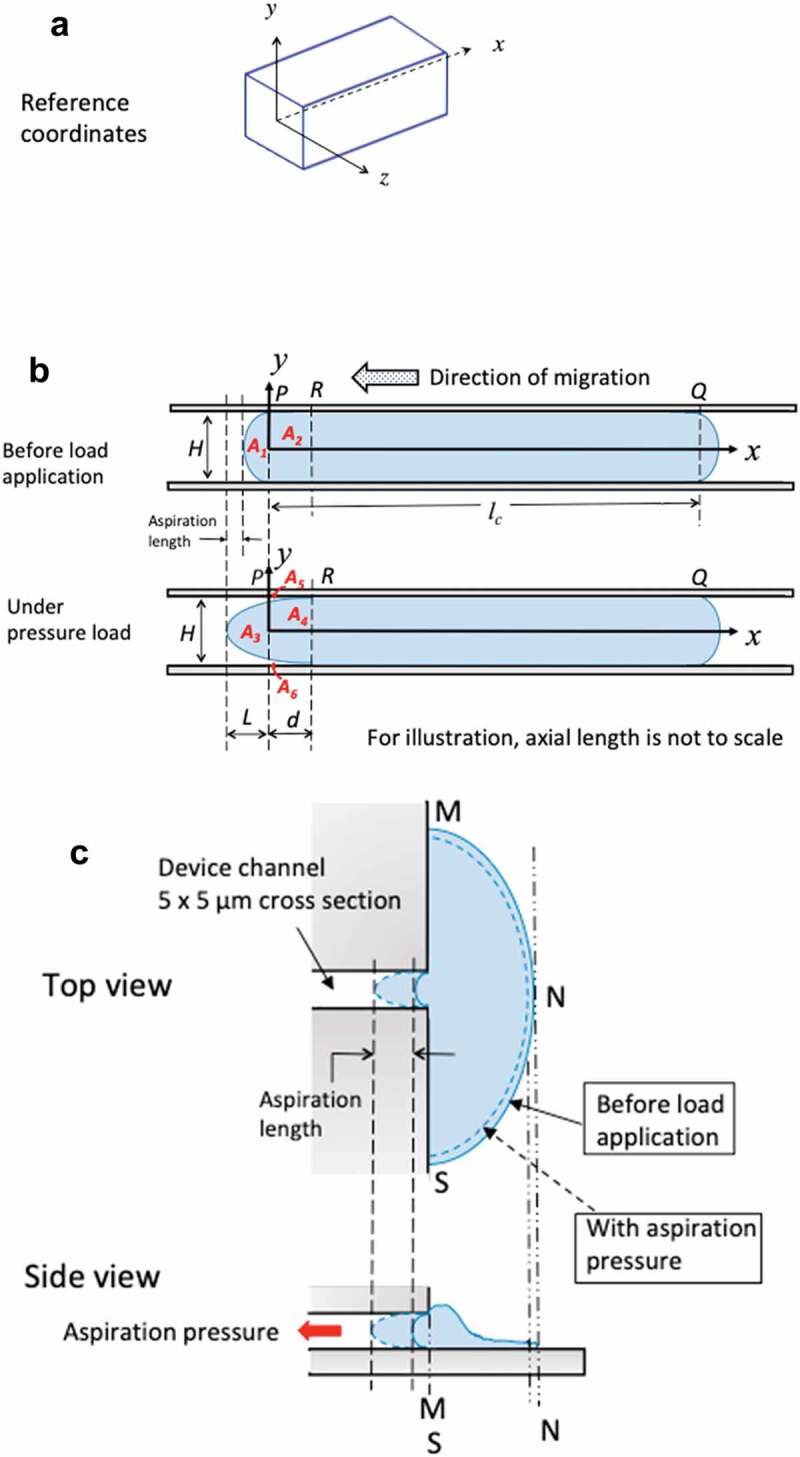
A) 3D reference coordinates for the channel. Channel has a cross section of 5 × 5 μm and an axial length of 530 μm. For illustration, the axial length of the channel is not to the scale. B) Simplified 2D plots on *xy* plane to illustrate the time-varying boundary conditions for the migrating cells before and when under aspiration pressure load. C) Top view and side view of GBMs from stationary group. Cells adhered to 2D reservoir substrate were in a stalling state with unsuccessful attempt to migrate into the microchannel of 5 × 5 μm in cross section

Simultaneous recording of the front and posterior-end displacements from actively migrating cells showed near-zero displacement at the posterior-end, confirming that recorded front-end displacements arose from the creep response of the migrating GBMs at the application of aspiration pressure load, excluding the likely sliding movement of the cell in the channel (Figure 6A). Corresponding recording from GBMs in stationary group showed movement of the posterior-end due to lamellipodium shrank as shown in Figure 6B when *p* = −20 cmH_2_O. To look into its effect on the apparent measurement of aspiration length, we found an effective radii of *R* = 12 \mum and *R*_shrink_ = 11.56 \mum from digitized video images before and after pressure load application (see Figures 7A–8C). Using an average height of *H_avg_ *= 1.5\mum for the lamellipodium adhering to the reservoir substrate (Bottier et al. 2011), we obtained an estimated volume loss of 16.5 $\mu m^{3}$ due to lamellipodium shrink on 2D substrate, from which we calculated an effective aspiration length $\Delta {\bar{U}}_{effective}$ of 0.66 \mum. It was subtracted from the apparent aspiration length measurement. Similar treatment was done for all cells in the stationary group as presented in Figure 4.

### Correlation with Actin and Myosin Distribution

Immuno-cytochemical examination showed a significantly higher level of intracellular actin and myosin-II filaments at both the front and posterior ends of GBMs in migrating group (Figure 7A). Distributions from the stationary group, on the other hand, showed low level of expression without regional preference. To compare the differences in two groups and to assess their regional differences in the degree of polarization, we expressed the fluorescent intensity along the axial length of the cell as a normalized factor with respect to their mean values averaged over the entire length (Figure 7 B, C). A factor higher than unity indicates a high degree of polarization of actin and myosin presence at the front and posterior ends of the GBMs in migrating group (Figure 7B). On the other hand, normalized intensity from the stationary group showed small oscillations around unity without regional differences (Figure 7C).

## Discussion

### Effects of migration in confinement and the intracellular actin/myosin distributions

Our results revealed that actively migrating GBMs, differ from those in stationary state, exhibited higher stiffness and with higher energy distribution in elastic storage over dissipative loss in their transient creep response. Intracellular distributions in actin and myosin from migrating group showed polarized distribution of actin and myosin at both the front and posterior ends of the cells, in contrast to the stationary group, which showed low-level expression of actin and myosin without any regional preference. Intracellular actomyosin contraction has been shown to play a major role in the force generation of cell migration in 3D microenvironment. Their contraction could cause regional buildup of intracellular pressure, contribute to elevated mechanical stiffness of the region. Further, intracellular pressure gradients could drive cytosol movement, cause altered rheological properties of the cytoplasm, contribute to the observed differences in creep response between these two states. Using a Voigt model, we characterized their differences in elastic response and the energy distribution in elastic storage vs. dissipative loss.

### Redistribution of actin and myosin when migrating in 3D confinement

Dynamic redistributions of cytoskeletal proteins, their network architecture, and the associated biochemical process could modulate regional compliance to facilitate the cell migration. Yanai et al. (Yanai et al. 1999) reported regional differences in stiffness and viscosity in migrating neutrophils on 2D substrate with lower stiffness and viscosity at the leading edge than the body and trailing edge. Their finding supports the description of mesenchymal mode cell migration on 2D substrate that consists of protrusion, adhesion at the leading edge, followed by contraction and release at the trailing edge governed by the interplay of three types of forces: cell-ECM adhesion, actin polymerization, and actomyosin contraction (Lämmermann and Sixt 2009). In 3D confinement, the interplay of these forces can alter significantly and a cell can migrate in the absence of cell-ECM attachment and actin polymerization, suggesting that the cell is propelled by intracellular hydrostatic pressure generated by actomyosin contraction (Balzer et al. 2012). Bui et al. (Bui et al. 2016) reported migrating GBMs exhibited different morphologies in microchannels of 5 × 5 μm and 15 × 15 μm cross section. In the former, cells occupied the entire channel space, derived tractions from 4 side walls. In the latter, GBMs attached to one or two adjacent channel walls, migrated in integrin-dependent mesenchymal mode as that on 2D substrate.

In this study, we considered migrating GBMs actively recruited actin/myosin contractile apparatus, contributed to the differences in mechanical and rheological properties as found. GBMs in stationary group were in a stalling state with part of cell body adhered to the substrate in the upstream reservoir outside of the channel. They responded to the aspiration pressure passively. Actively migrating GBMs, with elevated intracellular pressure, exhibited higher elastic stiffness at its front end that facilitate the establishment of traction on the sidewall needed for the forward movement through the anchoring effect.

Studies on isolated cells showed increased actomyosin contractility reduces cellular compliance. Kollmannsberger et al. (Kollmannsberger et al. 2011) observed non-linear increase in stiffness caused by the elevated internal tension of the cytoskeleton driven by the contractile activity of actomyosin. An et al. (An et al. 2002) demonstrated a reduction in localized compliance of a cell in the presence of a contractile agonist serotonin. With fibroblasts on 2D substrate, Kole et al. (Kole et al. 2005) showed migrating cells exhibit higher stiffness at the leading lamella and perinuclear region than stationary cells. From GBMs actively migrating in a 5 × 5 μm microfluidic channel, we found they exhibited different mechanical, rheological properties, and a polarized distribution in actin and myosin at the front and posterior ends (Figure 7), suggesting that the regional actomyosin contraction contributed to the regional higher intracellular pressure and the mechanical stiffness.

### Boundary conditions of GBM cells during aspiration

Migrating GBMs in confined microchannel. We first established 3D reference coordinates for the channel (Figure 8A) but chose to use simplified 2D plots on *xy*-plane to illustrate the time-varying boundary conditions for the migrating cells before and during the application of aspiration pressure. Before load application, with intracellular pressure the migrating GBM establishes surface contact with the channel sidewall from *P* to *Q* (having axial length *l_c_*) and having convex front and rear ends follows Laplace law (Figure 8B). That is, the entire surface from *P* to *Q* is displacement constrained. When under aspiration pressure, the GBM assumes a further convex front surface contour (Figure 8B). Consider conservation of mass (cytoplasma + membrane) for parts *A_1_* + *A_2_* in the initial state, and *A_3_* +* A_4_* in the loaded state, we should have:

(Eq.5) $$A_{1}+A_{2}\;=A_{3}+A_{4}\;from\;which\;\;\;A_{3}=A_{1}+\left(A_{2}-A_{4}\right)=A_{1}+\underset{two\;\;wedged\;\;areas}{\underbrace{A_{5}+A_{6}}}\;\;\;$$

That is, for non-zero measurements of aspiration lengths, part *A_1_* should acquire contributions *A_5_* and *A_6_* from part *A_2_* through streaming flow, implying membrane detachment from the sidewall as illustrated for segment *PR*. To validate it, we assumed the cellular front end follows a parabolic profile at the loaded state (Figure 8B):

(Eq. 6) $$\;\;\;\;\;\;\;\;\;\;\;\;\;\;\;\;\;\;\;\;\;\;\;\;\;\;\;\;x=\;\left(L+d\right)\;\;{\left[\frac{y}{\left(H\;/\;2\right)}\right]}^{2}\;-\;\;\;L\;\;\;$$

with$\;the\;boundary\;conditions:\;x\left(y=0\right)=-L\;\;\;\;\;and\;\;\;\;x\text{\textbackslash{}break}\left(y=\pm H\;/\;2\right)\;\;=\;\;d$. Here we define $\xi \;=\;\frac{y}{\left(H\;/\;2\right)}\;$ to account for the % coordinate from the mid-plane to the channel sidewall with $\;\left(1-\xi \right)\;$ measures the corresponding % detachment of *P*. To find the intercept of the protrusion with the *y*-axis, we calculated ξ correspond to

$$x(\xi )=\left(L+D\right)\;\xi {\;}^{2}-L=0$$

leading to

$$\xi^{2}=\frac{L}{L+D}.$$

Since both *L* and *d* are positive real numbers, it implies

$$\xi^{2}<1$$

and

ξ<1.

That is,

(1−ξ)>0

and *PR* has to detach from the channel wall to form two wedge areas *A5, A6*. Hence, the boundary conditions of constrained displacements from *P* to *R* were lost and the membrane segment was instead under instantaneous aspiration pressure load. That is, the boundary conditions converted from Dirichlet to Neumann type. Referencing the coordinates for the channel (Figure 8A), we wrote the initial boundary conditions for the migrating GBMs as

$$\begin{array}{rl} & \vec{u}\left(t=0,\;\;0\leq x\;\leq l_{c}\;,\;\;-\frac{H}{2}\leq y\leq \frac{H}{2\;},\;\;z=\pm \frac{W}{2}\right)\;=\vec{0}\\ & \vec{u}\left(t=0,\;\;0\leq x\leq l_{c},\;\;y=\pm \frac{H}{2}\;,\;\;-\frac{W}{2}\leq z\leq \frac{W}{2\;}\right)=\vec{0} \end{array}$$

with the notations: $\vec{u}=displacement\;\;veclor$, $l_{c}=the\;\;length\;\;of\;\;the\;\;migrating\;\;cell\;\;W=channel\;\;width,$ H=channelheight, noting that *H = W = *5 *μm.*

GBMs in stationary group. As explained in the previous sections, at the delivery of aspiration load, GBMs in stationary group shrank at their lamellipodium edges (from solid to dashed lined configuration in Figure 8C). At unloaded state (*t* = 0), the cell adhered to the substrate of the reservoir and all its contacting surface (the area confined in *M, N, S*) were displacement constrained ($\vec{u}\;=\vec{0}$). At the application of aspiration load on the cell protrusion in the channel, its lamellipodium edge shrank slightly with the majority of the cell body remained displacement constrained to the substrate to resist the aspiration load from the channel. Hence, there was a decrease in the surface area of the cell with constrained-displacements while the rest of the cell in the reservoir under a hydrostatic pressure (from the reservoir whose magnitude was negligible compared with the aspiration load).

In summary, both cell groups underwent time-varying displacement boundary conditions. Before load application, cells in two groups were in two different biological states: either to overcome the confinement to migrate forward or to stabilize themselves on 2D substrate in a stalling state with unsuccessful attempt to migrate into the confined channel. For each group, creep responses reflect the collective contributions from the underlying actin cortex, regional cytoskeletal networks, and their activities in the cytosol for the ‘intended’ biological function at the time. Our measurements of different creep responses revealed their corresponding differences in mechanical phenotypes.

Unlike conventional engineering materials, where time-dependent viscoelastic properties are studied to account for the intrinsic mechanical properties of the material and the tests are conducted with fixed boundary conditions independent of time. In this work, we applied the framework of Voigt creep model to evaluate the mechanical phenotypes of living GBM cells in two distinct states each with different intracellular cytoskeletal network, architecture, cytoplasma composition as well as their spatial distributions to meet the respective biological needs for the state. The use of aspiration pressure load in the creep test and the use of the framework from Voigt model provide an effective way to examine the differences in their mechanical phenotypes and biological responses to external mechanical stimuli.

### Any bleb formation

Separation of plasma membrane from its underlying cytoskeleton can result in the formation blebs occurring spontaneously in certain cell types due to elevated intracellular hydrostatic pressure (Charras et al. 2005; Tinevez et al. 2009; Strychalski and Guy 2016). Blebs has also been demonstrated in other cell types induced by the application of negative aspiration pressure arise from the relatively weaker attachment between the membrane and underlying actin cortex (Brugues et al. 2010; Campillo et al. 2012). In our current study, we didn’t observe any bleb formation when GBM cells, confined in 5 × 5 μm channel, were under prescribed negative pressure load up to 40 cmH_2_O. This is consistent with other studies using micropipette aspiration techniques on other cell types (Trickey et al. 2000; Zhou et al. 2010; Guo et al. 2012). It is likely that the occurrence of blebs is not observed across all cell types due to the variation of adhesion strengths between the actin cortex and the plasma membrane. Absence of specific genes in wild type cells could also trigger detachment in the membrane-cortex interface and can result in membrane protrusion when negative pressure is applied (Campillo et al. 2012) Studies on genetically targeted GBM cells could reveal the proteins responsible for the adhesion strength between the cytoskeleton and the plasma membrane. It is however beyond the scope of current study.

## Conclusion

While migrating in confined 3D microenvironment GBM cells exhibited different mechanical phenotypes than those at stationary state adhering to 2D substrate. At actively migrating state, GBMs exhibited higher mechanical stiffness with favored distribution in elastic energy storage over frictional loss. Cells in stationary group were in a stalling state with its majority of cell body adhere to 2D substrate and a small portion part of it in the microchannel. They were in a transitional state in their unsuccessful effort to migrate into the confined microchannel. Immuno-cytochemical studies showed polarized distributions in actin and myosin at the front and posterior ends of the migrating cells, suggesting regional intracellular pressure build up arise from acto-myosin contraction that contributed to the elevated stiffness and elastic energy storage to facilitate generation of traction and propulsive force to migrate in confinement. GBMs in quiescent state responded passively, exhibited higher compliance with favored flow behavior in part with the fluid movement into the cell body in the channel from the lamellipodium shrinkage on 2D substrate during the application of aspiration pressure load. Their immuno-cytochemical images showed no preferred regional distributions in actin and myosin, implying passive response from a relaxed state when under the application of mechanical load. Our study shed lights on the relevance of 2D mechanical assays when compared to 3D microenvironment in migration study. Our results suggest the cytoskeletal proteins kinetics plays a significant role in regional mechanical properties of the cell, which serves as major driving force in the alteration of the motility dynamics of a migrating cell. This study helps a better understanding of the migration biomechanics of cancerous cells and can serve as a platform for future studies investigating the biophysical properties associated with cancerous phenotypes of different cell types.
